# Targeted mutagenesis of the ryanodine receptor by Platinum TALENs causes slow swimming behaviour in Pacific bluefin tuna (*Thunnus orientalis*)

**DOI:** 10.1038/s41598-019-50418-3

**Published:** 2019-09-25

**Authors:** Kentaro Higuchi, Yukinori Kazeto, Yuichi Ozaki, Toshiya Yamaguchi, Yukinori Shimada, Yoshiaki Ina, Satoshi Soma, Yoshitaka Sakakura, Rie Goto, Takahiro Matsubara, Issei Nishiki, Yuki Iwasaki, Motoshige Yasuike, Yoji Nakamura, Aiko Matsuura, Shukei Masuma, Tetsushi Sakuma, Takashi Yamamoto, Tetsuji Masaoka, Takanori Kobayashi, Atushi Fujiwara, Koichiro Gen

**Affiliations:** 1 Seikai National Fisheries Research Institute, Japan Fisheries Research and Education Agency, Nagasaki, 851-2213 Japan; 2 Kamiura Station, National Research Institute of Aquaculture, Japan Fisheries Research and Education Agency, Saiki, Oita 879-2602 Japan; 3 National Research Institute of Aquaculture, Japan Fisheries Research and Education Agency, Watarai, Mie 519-0423 Japan; 4 Organization for Marine Science and Technology, Graduate School of Fisheries and Environmental Sciences, Nagasaki University, Nagasaki, 852-8521 Japan; 5 Nishiura Station, South Ehime Fisheries Research Center, Ehime University, Minamiuwa, Ehime 798-4206 Japan; 6 National Research Institute of Fisheries Science, Japan Fisheries Research and Education Agency, Yokohama, Kanagawa 236-8648 Japan; 7 Aquaculture Research Institute, Kindai University, Nishimuro, Wakayama 649-2211 Japan; 8 Department of Mathematical and Life Sciences, Graduate School of Science, Hiroshima University, Higashi-Hiroshima, Hiroshima 739-8526 Japan; 9 Headquarters, Japan Fisheries Research and Education Agency, Yokohama, Kanagawa 220-6115 Japan

**Keywords:** Genetic engineering, Mutagenesis

## Abstract

In bluefin tuna aquaculture, high mortalities of hatchery-reared juveniles occur in sea cages owing to wall collisions that are caused by high-speed swimming in panic due to changes in illuminance. Here, we report that targeted gene mutagenesis of the ryanodine receptor (RyR1b), which allows the sarcoplasmic reticulum to release Ca^2+^ in fast skeletal muscle, using highly active Platinum TALENs caused slow swimming behaviour in response to external stimuli in Pacific bluefin tuna (PBT) larvae. This characteristic would be a useful trait to prevent wall collisions in aquaculture production. A pair of Platinum TALENs targeting exons 2 and 43 of the PBT *ryr1b* gene induced deletions in each TALEN target site of the injected embryos with extremely high efficiency. In addition, *ryr1b* expression was significantly decreased in the mutated G0 larvae at 7 days after hatching (DAH). A touch-evoked escape behaviour assay revealed that the *ryr1b*-mutated PBT larvae swam away much less efficiently in response to mechanosensory stimulation at 7 DAH than did the wild-type larvae. Our results demonstrate that genome editing technologies are effective tools for determining the functional characterization of genes in a comparatively short period, and create avenues for facilitating genetic studies and breeding of bluefin tuna species.

## Introduction

Bluefin tuna aquaculture is of high commercial value and is widely undertaken in many countries including Mexico, Australia, and Japan, as well as in the Mediterranean Sea^1^. The majority of aquaculture businesses are dependent on wild-caught juveniles for seed stocks, and negative influences of this dependency on the management of wild tuna stocks have been reported^2,3^. To sustain tuna aquaculture and preserve wild stocks, a closed-cycle aquaculture system must be established. However, high mortality often occurs after hatchery-reared juveniles (50‒70 mm in total length) are transferred from mass-culture tanks to sea cages for grow-out, with mortalities reaching over 80% from cage stocking to the next year^4^. A major cause of mortality is collision with walls due to high-speed swimming in panic induced by sudden changes in light intensity such as lightning, the lamplights of fishing boats near the tuna culture cages, and during dawn and dusk^5–7^. The mortality due to wall collision continues until the fish reach marketable size (50‒70 kg in body weight), resulting in considerable economic losses to the fish farmer.

High-speed swimming is associated with muscle contraction in skeletal muscle. Generally, once motor neurons in each side of the spinal cord are activated by output from the central nervous system (CNS), motor terminals release acetylcholine at the neuromuscular junction to depolarise the muscle membrane^8,9^, and this change in membrane potential is converted to muscle movement by excitation-contraction (E-C) coupling^10^. In E-C coupling, depolarisations of the plasma membrane spread down the transverse-tubules (t-tubules) and cause conformational changes of the sarcolemmal voltage-gated Ca^2+^ channel (Ca_V_1.1) in the t-tubule membrane^11^. Ca_V_1.1 then triggers the opening of the ryanodine receptor 1 (RyR1) in the adjacent sarcoplasmic reticulum (SR) to allow Ca^2+^ release from the SR to the cytosol^12^. Elevated cytoplasmic Ca^2+^, in turn, activates the sliding of actin/myosin to produce muscle contractions. Recently, a *relatively relaxed* zebrafish mutant was identified to have undergone a spontaneous mutation and displayed slow swimming in response to external stimuli owing to weak muscle contractions despite normal output from the CNS^13^. The zebrafish mutants carried a 32-bp cDNA insertion including a nonsense codon between exon 48 and 49 of their *ryr1b* mRNA, which encoded RyR1 that is dominantly expressed in the fast skeletal muscle (glycolytic musculature used for burst activity)^13^, thus suggesting that *ryr1b* is the key gene involved in the high-speed swimming of fish.

Recent advances in genome editing technology, transcriptional activator-like effector nucleases (TALENs) and clustered regularly interspaced short palindromic repeats associated with Cas9 (CRISPR/Cas9), have led the way for genomic engineering in different fish species for basic functional studies as well as applied research in aquaculture^14,15^. The TALENs and CRISPR/Cas9 allow for the generation of bi-allelic mutants with clear phenotypes in the G0 generation without having to cross animals for several generations^16–18^. The CRISPR/Cas9 is more cost effective, convenient and efficient to be assembled, which allows for large-scale and high-throughput genome modification in many fish species^19^; however, due to the short recognition site of only 18 nucleotides, off-target effect may occur^20^. On the other hand, the advantage of TALEN technology is its high specificity of sequence recognition compared with the CRISPR/Cas9^21^. The TALEN can typically recognize 15- to 20-bp DNA sequence for the left and right monomer each; thus, a total of 30- to 40-bp sequence is specifically recognized, which provides much higher specificity and much less possibility introducing off-targets into the genome than the CRISPR/Cas9 system^22^. Moreover, the TALENs was unlikely to induce mutations when the spacers between two binding sites were >24 bp long^22^. These recognition mechanisms account for the minimal off-target effects of this technology. In addition, a recent study demonstrated that TALENs with variable repeats harbouring non-repeat-variable di-residue (non-RVD) variations, called Platinum TALENs, showed higher activity than TALENs with constant repeats without non-RVD variations in cells, frog, and rat embryos^23^. In the present study, we successfully produced Pacific bluefin tuna (PBT, *Thunnus orientalis*) larvae exhibiting slow swimming in response to tactile stimuli due to mutations of the *ryr1b* gene induced by Platinum TALENs. The slow swimming characteristics are a useful trait for the prevention of wall collisions in bluefin tuna aquaculture. These results suggest that the Platinum TALEN system is an effective genome editing tool for breeding new varieties with valuable traits in bluefin tuna.

## Results

### *ryr1b* is expressed by the fast muscle of wild-type PBT larvae

**Figure 1:**
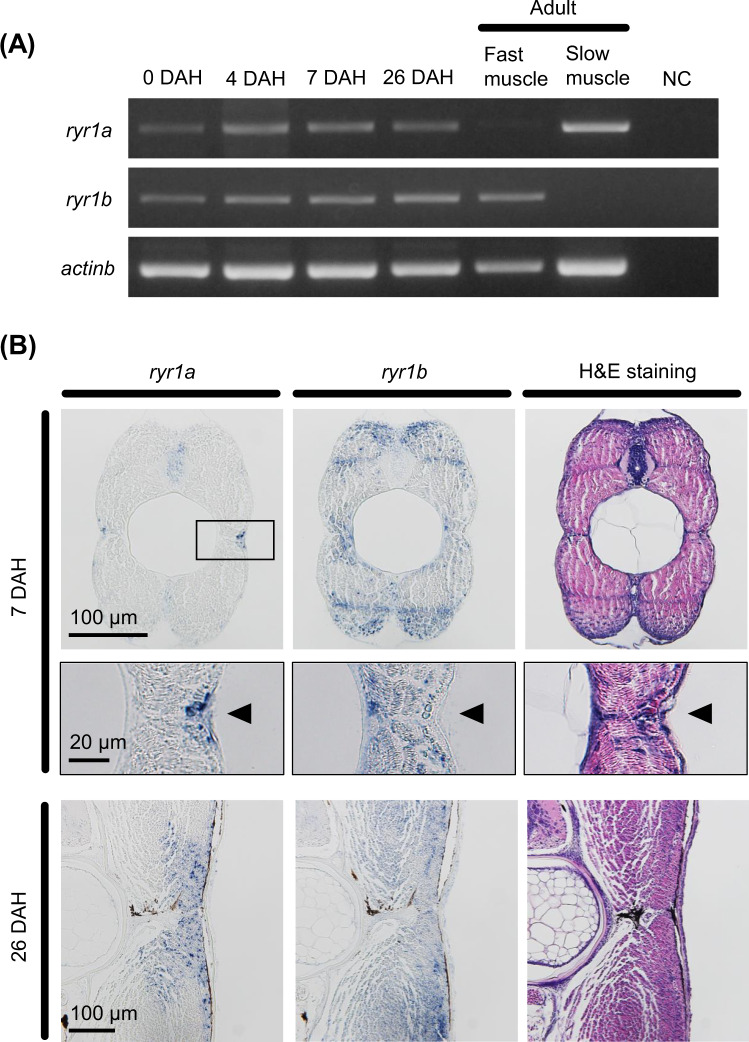
r*yr1a* and *ryr1b* expression by fast and slow muscle in wild-type Pacific bluefin tuna, respectively. (**A**) RT-PCR analysis in larvae at 0, 4, 7, and 26 days after hatching (DAH), and in adult fast and slow muscle. RT-PCR shows that both mRNA are detected from hatching larvae to adulthood, and *ryr1a* and *ryr1b* genes specifically express in adult slow and fast muscle, respectively. (**B**) The distribution of *ryr1a* and *ryr1b* mRNA in the developing skeletal muscle of larvae at 7 and 26 DAH by *in situ* hybridisation. The insets indicate higher magnification of the slow developing muscle area. Cross-sections show that *ryr1a* is expressed by the lateral muscle of the vertebral column-slow developing muscle (arrowheads), whereas *ryr1b* is expressed by a large proportion of the muscle-both fast and slow developing muscles.

Gene expression and the localisation of *ryr1a* and *ryr1b* in larval and adult stage muscle of wild-type PBT were examined. RT-PCR showed that both *ryr1a* and *ryr1b* expression was detected from 0 to 26 days after hatching (DAH) through to adulthood (Fig. 1A). Results also showed that *ryr1b* expression, but not *ryr1a* expression, was detected by RT-PCR in adult fast muscles (Fig. 1A). However, *in situ* hybridisation in 7- and 26-DAH larvae revealed that *ryr1b* was expressed by a large proportion of skeletal muscles including the slow developing skeletal muscle where *ryr1a* mRNA was detected (Fig. 1B).

### Platinum TALENs effectively induce mutations in the *ryr1b* gene of PBT

**Figure 2:**
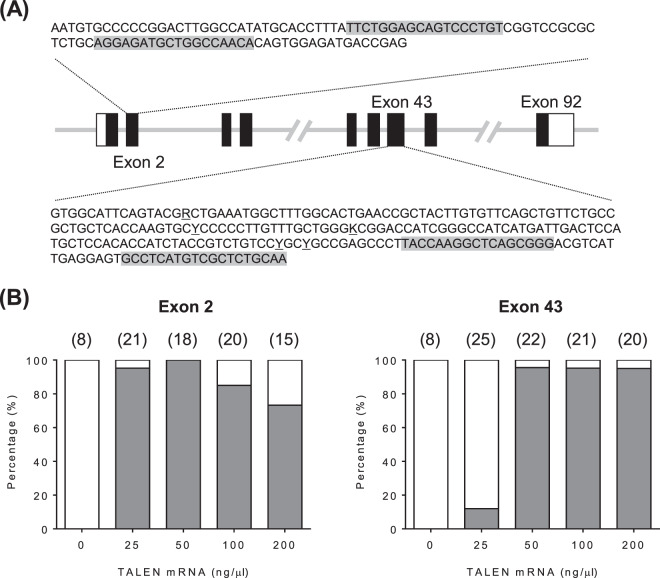
Design of *ryr1b*-TALENs and mutation rates of Pacific bluefin tuna embryos injected with Platinum TALENs. (**A**) *ryr1b*-TALENs designed to target exons 2 and 43 of the gene. The solid and open boxes represent coding and untranslated exon regions, respectively. The grey boxed sequences indicate the TALEN binding sites. (**B**) Mutation rates of the TALEN-injected hatched embryos analysed by heteroduplex mobility assays (HMA). The grey and white boxes represent the proportion of positive and negative embryos by HMA. The number of analysed embryos is indicated in brackets.

Platinum TALENs were designed to target exons 2 and 43 of the PBT *ryr1b* gene (Fig. 2A). To evaluate the toxicity and activity of the TALENs, we injected different doses of the TALEN mRNA pair (25, 50, 100, 200, and 400 ng/µl of each left and right TALEN mRNA) into PBT embryos at the 1- to 4-cell stage. After 40 h, the number of normally hatched (not malformed) embryos with straight bodies was counted. In the *ryr1b-ex2* TALEN, the normally hatching rates of embryos injected with 100‒400 ng/µl of TALEN mRNA were considerably lower than those of embryos not injected and those injected with 25 and 50 ng/µl, although injection with 100 ng/µl of TALEN did not show significant toxicity in Experiment 3 (Table 1). In the *ryr1b-ex43* TALEN, high toxicity was observed for embryos injected with 400 ng/µl of TALENs (Table 1). Injection with 25‒200 ng/µl of mRNA had no adverse effects on the percentages of normally hatched embryos in Experiments 2 and 3 (Table 1). To examine the mutation rates of the injected embryos, genomic DNA was prepared from individual TALEN-injected or not injected embryos at the hatching stage in Experiment 3, and was analysed using a heteroduplex mobility assay (HMA) for the presence of DNA sequence variants at the targeted sites (Supplementary Figs S1 and S2). Nearly all TALEN RNA-injected embryos had targeted mutations, except for the embryos injected with 25 ng/µl of *ryr1b-ex43* TALEN (Fig. 2B). Together, the 50 ng/µl concentration of *ryr1b-ex2* TALEN and 200 ng/µl concentration of *ryr1b-ex43* TALEN were the best concentrations, which led to high survival and mutation rates of the injected embryos. Sequence analysis of PCR amplicons covering the targeted sites in the embryos injected with TALENs at various concentrations indicated the induction of a spectrum of indel mutation, consistent with what is expected from non-homologous end-joining (Supplementary Figs S3 and S4).

**Table 1:** Survival rates of Pacific bluefin tuna embryos injected with TALENs.

TALENs	Trial	mRNA concentration (ng/µl each pair)	No. of injected embryos	% of hatched embryos^a^	% of normally hatched embryos^b^
Exon 2	Exp. 1	400	32	6.3	3.1
		0	30	100.0	96.7
	Exp. 2	200	31	67.7	0.0
		100	30	83.3	0.0
		50	31	87.1	61.3
		0	30	73.3	73.3
	Exp. 3	200	29	51.7	27.6
		100	42	88.1	73.8
		50	30	86.7	83.3
		25	34	70.6	55.9
		0	45	97.8	97.8
Exon 43	Exp. 1	400	28	60.7	3.6
		0	30	96.7	90.0
	Exp. 2	200	38	92.1	57.9
		100	20	100.0	90.0
		50	43	95.3	74.4
		0	50	100.0	72.0
	Exp. 3	200	35	80.0	71.4
		100	30	93.3	86.7
		50	31	96.8	87.1
		25	33	90.9	66.7
		0	45	95.6	95.6

^a^Percentages of hatched embryos per injected embryos.

^b^Percentages of normally hatched (not malformed) embryos per injected embryos.

### Reduction of *ryr1b* gene expression in *ryr1b*-mutated PBT larvae

**Figure 3:**
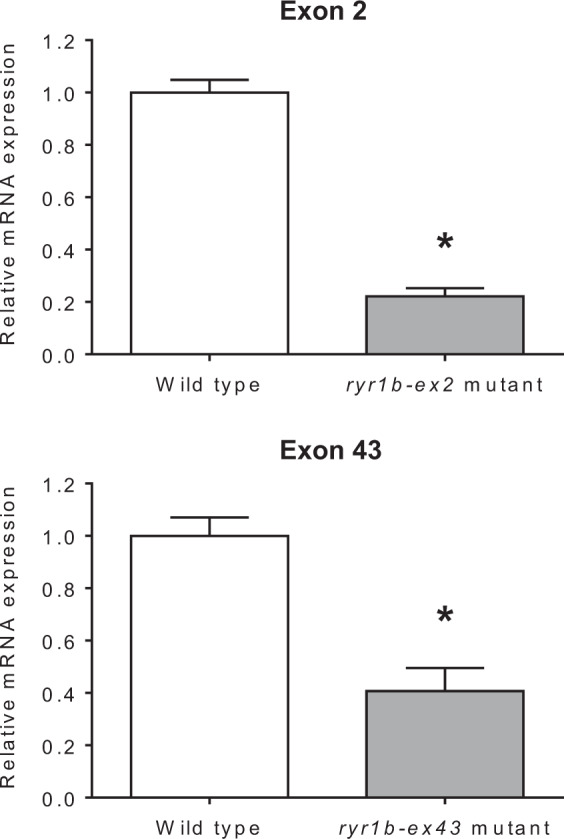
Gene expression of ryanodine receptor in *ryr1b*-mutated Pacific bluefin tuna. Relative mRNA expression of *ryr1b* in *ryr1b*-mutated and wild-type larvae at 7 days after hatching. The data represent means ± SEM (*n* = 10 fish). Significant differences between *ryr1b*-mutated and wild-type larvae are indicated by the asterisks (*P* < 0.05).

The effects of the indel mutations in exons 2 or 43 of the *ryr1b* gene were evaluated at the mRNA levels. The best concentrations of *ryr1b-ex2* (50 ng/µl) or *ryr1b-ex43* TALEN (200 ng/µl) were injected into 1-cell stage embryos. Normal hatched embryos with each TALEN injection and non-injection (wild-type) were separately transferred into 10-L kreisel tanks, and reared with rotifers (*Brachionus plicatilis*) at 24 °C. Analyses of *ryr1b* gene expression of mutant larvae at 7 DAH revealed lower transcript abundance than that of samples from the wild-type (Fig. 3). All larvae used in the qPCR assay were genotyped by HMA on genomic DNA isolated from the caudal fins, and were found to carry mutations at the targeted sites.

### *ryr1b*-mutated PBT larvae exhibit a slow touch-evoked escape response

**Figure 4:**
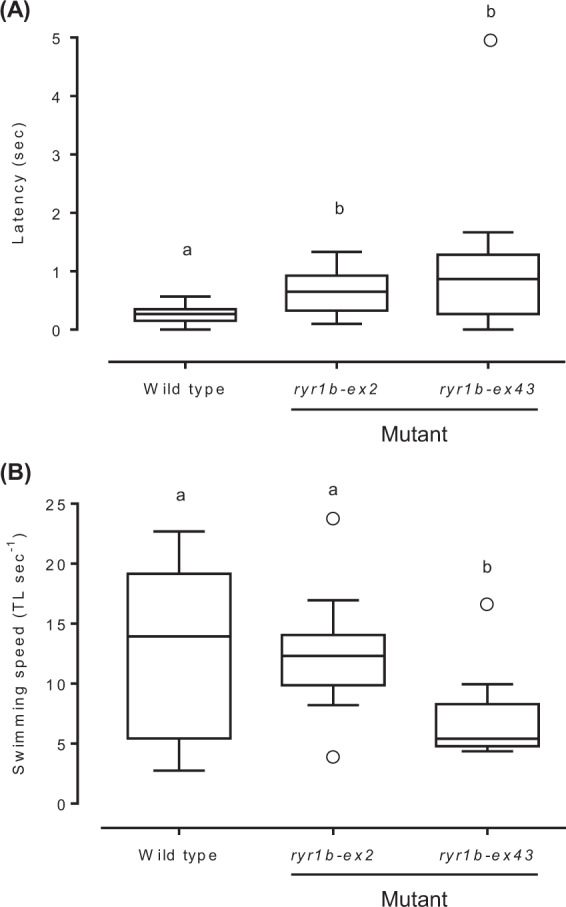
*ryr1b*-mutated larvae exhibit a slow touch-evoked escape response. (**A**) Latency period between touch stimulation and escape response, and (**B**) relative swimming speed during escape response in wild-type and *ryr1b*-mutated larvae at 7 days after hatching. Boxplots show 25th‒75th percentiles (*box*) with median (*line*), with points outside the box representing outliers (*n* = 12, wild type; *n* = 10, *ryr1b-ex2* and *ryr1b-ex43* mutants). *Whiskers* represent maximum and minimum values. Significant differences are indicated by different letters (*P* < 0.05).

At 7 DAH, the swimming phenotypes of G0 larvae were examined using a touch-evoked escape behaviour assay and a digital camera. Typically, wild-type larvae swam away rapidly upon tactile stimulation using dissecting needles, whereas *ryr1b*-mutated larvae swam away much less efficiently (Supplementary Movies 1). The latent period between the touch stimulation and escape response was significantly longer in *ryr1b*-mutated larvae against both exons than that in their wild-type siblings (Fig. 4A). In addition, the swimming speed during the escape response was significantly lower in *ryr1b-ex43* mutated larvae than that in the wild-type larvae (Fig. 4B). These observations indicate that *ryr1b*-mutated PBT larvae exhibited impaired swimming response to mechanosensory stimulation.

## Discussion

In the present study, we successfully established a Platinum TALEN system to induce targeted mutations with high efficiency in the PBT. In addition, we showed that *ryr1b*-mutated PBT in the G0 generation exhibited slow swimming characteristics, which would be a useful trait in aquaculture to prevent wall collisions. These results indicate that genome editing technologies are effective tools for studying gene functions, as well as creating new genetically modified strains with special characteristics in PBT aquaculture. To date, genetic improvement in bluefin tuna species that have long generation times (3‒5 years) has been challenging because it takes a long time to produce F2 individuals of a single double allelic mutation^2^. In addition, technology for selective mating by artificial insemination has not been established because mature bluefin tuna are very difficult to handle owing to their large body size and very delicate skin, which can be easily damaged^24^. Our results demonstrated that the Platinum TALEN system induced site-specific mutation with high efficiency, suggesting that homozygous null mutants could be generated in the F1 generation by crossing between the TALEN-injected G0 founders^17^. In future, this system would allow precise changes in the genome composition based on the aquaculture requirements, thereby allowing for precision breeding within a comparatively short period without selective mating in the PBT.

The *ryr1a* was expressed by slow muscles and *ryr1b* was expressed by fast muscles in adult PBT. This differential expression of the *ryr1* genes in slow and fast muscles appears in other teleosts, such as the zebrafish^25^, blue marlin (*Makaira nigricans*)^26^, sardine (*Sardina pilchardus*)^27^ and yellowfin tuna (*T*. *albacares*)^28^. However, our study used *in situ* hybridisation to reveal that *ryr1b* was expressed in both types of developing skeletal muscles at 7 and 26 DAH. Thus, PBT *ryr1b* mRNA expression is localised to both fast and slow developing skeletal muscles during larval and juvenile stages, and is confined to the fast muscle in the adult stage. In a previous study, applying *ryr1* probes and F59 antibody, a marker of slow muscle, to developing embryos of double-labelled zebrafish showed that *ryr1a* mRNA co-localised exclusively with slow muscle staining, whereas *ryr1b* expression was observed throughout both muscle types^29^. On the other hand, in 7-DAH PBT larvae *ryr1b* is expressed by a large proportion of a skeletal muscles, whereas *ryr1a* mRNA expression was localised to specific small regions of the lateral muscle of the vertebral column. These results suggest that fast skeletal muscle might be dominant in swimming behaviour of larval stage PBT.

In the present study, a touch-evoked escape behaviour assay demonstrated that *ryr1b*-mutated PBT swim away much less efficiently in response to mechanosensory stimulation at 7 DAH than did the wild-type larvae. Moreover, basic *ryr1b* mRNA levels were significantly reduced in PBT mutants. RyR1 is a skeletal muscle intracellular Ca^2+^ channel that plays a central role in the rapid release of Ca^2+^ from intracellular stores into the cytosol and is essential for numerous cellular functions including E-C coupling^30^. In addition, it is well known that the function of RyR1 is highly conserved in vertebrates from mammals to fish^13,28,30^. The *ryr1b* zebrafish mutants exhibiting slow swimming in response to touch have shown that Ca^2+^ transients in the muscle cytosol were dramatically decreased in the fast muscles^13^. Thus, our results suggest that the functional deficiency of RyR1b leads to weak muscle contraction owing to a decrease in Ca^2+^ released from the SR to the cytosol, followed by impaired response to touch in the PBT larvae. Although the *ryr1b* zebrafish mutants did not show a significant increase in latency period of the response to touch^13^, the disagreement with our results may be due to differences in the slow and fast skeletal muscle composition. Conversely, even when the t-tubules and SR were not damaged in the *ryr1b* zebrafish mutants, there was a dramatic decrease in the fast muscle Ca_V_1.1^13^, which triggers the opening of RyR1 to release Ca^2+^ for myofibril contraction^11^. A cytoplasmic domain of RyR1 is essential for physical interaction with an intercellular loop of Ca_V_1.1 in cultured mammalian myotubes^31,32^. Although we did not determine the expression levels of fast muscle CaV1.1 in the PBT mutants, the presence of RyR1 may also be required for the expression and correct localization of CaV1.1 in the calcium releasing units of skeletal muscle of the PBT^11^.

The swimming speed of *ryr1b-ex43* mutated PBT during the escape response was slower than that of the wild-type larvae. At present, we lack explanation for this decrease in swimming speed in *ryr1b*-mutated PBT. One possibility is that a reduction in RyR1b expression may lead to a morphological defect in the fast muscle. In a previous study, electron microscopy analysis of the zebrafish *ryr* mutant muscles clearly demonstrated amorphous cores in the fast muscle fibres, which share a crucial feature with human multi-minicore disease (MmD)^13^. In contrast, the morphological defects in the fast muscle are likely to have a significant impact on the development and maintenance of the muscle itself, which leads to a reduction in growth and/or in fillet quality in aquaculture. Future studies are required to examine whether *ryr1b*-mutated PBT displayed ultrastructural defects similar to those seen in MmD muscles.

It has been reported that there are potential off-target alternations in the CRISPR/Cas9 mediated genome editing^20^. However, previous reports indicated that there were few or no off-target alternations in pre-selected candidate sites harbouring 2- or fewer-bp mismatches in the 12-bp of the targeting sequence followed by NGG protospacer adjacent motif in mice, *Drosophila*, *C*. *elegans*, and fish^33,34^. Meanwhile, because the DNA-binding domain in each TALEN has a sufficiently long binding sequence that exhibits high DNA-binding specificity, it is likely that there are few off-target effects caused by mutagenesis of potential off-target sites of the TALENs^17^. In fact, previous *in vivo* experiments in fish identified no mutation at potential off-target sites with significant similarity to on-target site^17^. Moreover, in this study, the *ryr* mutants generated by two different Platinum TALENs showed the similar swimming phenotypes. Thus, although we did not examine alternations at potential off-target sites in *ryr1b*-mutated PBT, the slow swimming phenotypes presented here are not likely to be derived from off-target effects.

In conclusion, we have successfully applied a genome editing technology to bluefin tuna species for the first time. Moreover, our studies demonstrated that *ryr1b*-mutated PBT exhibited slow swimming in response to mechanosensory stimulation, which may be caused by weak muscle contractions due to a decrease in Ca^2+^ transients in the fast muscle cytosol. In PBT aquaculture, slow swimming is a valuable trait to prevent wall collisions because collisions are caused by high-speed swimming in panic that is induced by sudden light changes^7^. In future, further studies are needed to examine the response to sudden illumination of *ryr1b*-mutated PBT in juvenile and later stages. On the other hand, RyR1 mutations in humans lead to a congenital recessive myopathy, which is defined by amorphous cores in muscle^35^. Although RyR1-deficient mice and zebrafish die on the day of birth and 7‒15 days post fertilization, respectively^13,36^, the *ryr1b*-mutated PBT survived to a month after hatching (data not shown). Moreover, morphological defects including amorphous cores in muscle may affect the fillet quality. Furthermore, slow swimming behaviour may negatively impact spawning behaviour and mating success because during spawning events several males aggressively chase one mature female repeatedly with high speed^37^. Therefore, it is necessary to raise *ryr1b*-mutated PBT to adulthood for the establishment of homozygous null F1 fish to examine economically important traits especially growth performance and fillet quality as well as the survival. These studies promote the genetic engineering of the PBT, with genome editing technologies having the potential to improve the qualities and economic value of the PBT for future aquaculture.

## Materials and Methods

### Embryo preparation

Adult PBT were reared in land-based tanks (20 m diameter, 6 m depth) at Seikai National Fisheries Research Institute (SNFRI), Japan Fisheries Research and Education Agency (FRA) (Nagasaki, Japan), and circular sea cages (30 m diameter, 15 m depth) at Amami Station, Aquaculture Research Institute, Kindai University (Kagoshima, Japan). Fertilised eggs were obtained using a net immediately after spontaneous spawning in the tanks or cages, and then moved into the laboratory within 10 min. All animal care and procedures were performed in accordance with the Guidelines for Animal Experimentation SNFRI, FRA. All experiments were approved by the Animal Research Committee of SNFRI.

### *in situ* hybridisation

Riboprobe synthesis, sectioning and *in situ* hybridisation were performed as described previously^38^. Digoxigenin (DIG)-labelled anti-sense RNA probes were individually synthesized from corresponding open reading frames: *ryr1a*, nucleotides 12,904‒14,062 (1,178 bps); *ryr1b*, nucleotides 11,508‒12,548 (1,041 bps) (Supplementary Table S1).

### Design and construction of *ryr1b*-TALENs

The TALEN target sites of PBT *ryr1b* are located in exons 2 and 43 (Fig. 1A). The protocol for TALEN assembly has been previously described^23^. TALEN mRNA was transcribed *in vitro* using a mMESSAGE mMACHINE™ T7 ULTRA Transcription Kit (Thermo Fisher Scientific, Waltham, MA). The resultant mRNA was purified by phenol:chloroform extraction and isopropanol precipitation following the manufacturer’s protocol and finally resuspended in RNase-free water at various concentrations (25, 50, 100, 200 and 400 ng/µl) for each TALEN.

### Microinjection

The microinjection of *ryr1b*-TALENs was performed using a needle (G-1, Narishige, Tokyo, Japan) with an added constriction (3‒5 μm inner diameter) in the upper part of the tip in order to control the pressure required for injecting the RNA solution into the embryo and to prevent backflow of cytoplasm from the embryo as described previously^39–41^. Embryos in the 1‒4 cell stage were placed on a 1% agar-coated Petri dish filled with 50% sterilised seawater, and then microinjected with TALEN mRNA. The injected embryos were cultured in a 6-well plate filled with sterilised seawater containing antibiotics at 24 °C until hatching.

### HMA

The efficiency of the target mutations in the injected embryos was examined by HMA^42^. Briefly, genomic DNA samples were extracted from individual embryos during the hatching stage using a DNeasy Blood & Tissue Kit (QIAGEN GmbH, Dusseldorf, Germany). Genomic regions containing the TALEN target sites were amplified using specific primer sets (Supplementary Table S2). The PCR cycling parameters were as follows: 35 cycles of 30 sec at 94 °C, 30 sec at 60 °C, and 30 sec at 72 °C. To detect heteroduplex in the amplicons, the PCR products were analysed using a microchip electrophoresis system, MCE-202 MultiNA, (Shimadzu, Kyoto, Japan) with a DNA-500 reagent kit (Shimadzu).

### Amplicon sequencing of mutated target sites

The genomic DNA was extracted as described above, and the target region was amplified using the 2-step tailed-PCR method essentially described in Forshew *et al*.^43^. PCR products were purified with Agencourt AMPure XP beads (Beckman Coulter, Brea, CA), and sequenced on a NextSeq. 500 platform (Illumina, San Diego, CA) with paired-end reads of 151 base pairs according to the manufacturer’s protocol.

Raw next-generation sequencing reads were filtered and trimmed using Trimmomatic v. 0.3641^44^ with CROP:145, LEADING:30, TRAILING:20, SLIDINGWINDOW:4:20, and MINLEN:100 options, and merged using the VSEARCH v 2.0.4 software program^45^ with fastq_mergepairs option. The merged reads were mapped to the reference sequence of the PBT^46^ using the BWA-MEM v 0.7.12 software program^47^. The mapped reads were realigned using RealignerTargetCreator and IndelRealigner in the Genome Analysis Toolkit v 3.6 software program^48^ and the aligned sequences of the target region were obtained from the mapped reads and CIGAR information in the SAM file.

### qPCR

Total RNA was extracted from individual larvae at 7 DAH using ISOGEN II (NIPPON GENE, Toyama, Japan) and the total RNA was treated using TURBO DNase (Ambion, Austin, TX) as specified by the manufacturer’s protocol. A total of 0.5 µg of total RNA was reverse-transcribed using the Omniscript RT kit (QIAGEN GmbH), after priming with random hexamers. The assay was run on a Light cycler 480 (Roche Diagnostics, Mannheim, Germany), with the following thermal cycling conditions: 95 °C for 10 min, followed by 45 cycles of 95 °C for 10 sec and 58 °C for 30 sec. The reaction volumes (10 µl) contained 2.5 µl of a 1/40 dilution of the final cDNA, 0.5 µM of the forward and reverse primers, and 5 µl of FastStart Essential DNA Green Master (Roche Diagnostics). Primers for the amplicons are listed in Supplementary Table S3. We used a plasmid containing a partial cDNA sequence of a target gene as the standard for quantification. The mean value of the wild-type was set to 1 to improve the presentation of results. In consideration of potential off-target mutations of reference genes, results were not normalized using reference gene. Technical duplicates were run for all experimental samples and standards.

### Touch-evoked escape behaviour assay

The touch-evoked response was measured in the 7-DAH mutated and wild-type larvae as described previously^49^. One larva at a time was placed on a Petri dish filled with sterilised seawater. Mechanosensory stimuli were delivered to the side of the embryo bodies using dissecting needles. Videos were captured with a digital camera (Sony, Tokyo, Japan) and were analysed with DIPP-Motion V/3D (DITECT, Tokyo, Japan). Swimming speed was calculated using the distance travelled in a sec. The swimming speed was standardized by the fish total length in consideration of the size difference of individual larvae.

### Statistical analysis

Data are presented as the mean ± standard error of the mean, except for the data of the touch-evoked escape assay that are presented as the median. Statistical significance was analysed with one-way analysis of variance followed by Tukey’s multiple comparison test. Data of latency were subjected to Steel-Swass nonparametric multiple comparison test. The statistical significance level was determined at the *P* < 0.05 level using Prism 6.0 (GraphPad Software, San Diego, CA) and R V.3.0.1 software program.

## Supplementary information

Supplementary Figure

Supplementary Movie 1

Supplementary Movie 2
