# Author Correction: Targeted mutagenesis of the ryanodine receptor by Platinum TALENs causes slow swimming behaviour in Pacific bluefin tuna (*Thunnus orientalis*)

**DOI:** 10.1038/s41598-020-65964-4

**Published:** 2020-06-04

**Authors:** Kentaro Higuchi, Yukinori Kazeto, Yuichi Ozaki, Toshiya Yamaguchi, Yukinori Shimada, Yoshiaki Ina, Satoshi Soma, Yoshitaka Sakakura, Rie Goto, Takahiro Matsubara, Issei Nishiki, Yuki Iwasaki, Motoshige Yasuike, Yoji Nakamura, Aiko Matsuura, Shukei Masuma, Tetsushi Sakuma, Takashi Yamamoto, Tetsuji Masaoka, Takanori Kobayashi, Atushi Fujiwara, Koichiro Gen

**Affiliations:** 1 Seikai National Fisheries Research Institute, Japan Fisheries Research and Education Agency, Nagasaki, 851-2213 Japan; 2 Kamiura Station, National Research Institute of Aquaculture, Japan Fisheries Research and Education Agency, Saiki, Oita 879-2602 Japan; 3 National Research Institute of Aquaculture, Japan Fisheries Research and Education Agency, Watarai, Mie 519-0423 Japan; 4 Organization for Marine Science and Technology, Graduate School of Fisheries and Environmental Sciences, Nagasaki University, Nagasaki, 852-8521 Japan; 5 Nishiura Station, South Ehime Fisheries Research Center, Ehime University, Minamiuwa, Ehime 798-4206 Japan; 6 National Research Institute of Fisheries Science, Japan Fisheries Research and Education Agency, Yokohama, Kanagawa 236-8648 Japan; 7 Aquaculture Research Institute, Kindai University, Nishimuro, Wakayama 649-2211 Japan; 8 Department of Mathematical and Life Sciences, Graduate School of Science, Hiroshima University, Higashi-Hiroshima, Hiroshima 739-8526 Japan; 9 Headquarters, Japan Fisheries Research and Education Agency, Yokohama, Kanagawa 220-6115 Japan

Correction to: *Scientific Reports* 10.1038/s41598-019-50418-3, published online 25 September 2019

This Article contains an error in the Methods section under subheading ‘Embryo preparation’.

“All experiments were approved by the Animal Research Committee of SNFRI”

should read:

“All experiments were approved by the Animal Experiment Committee of SNFRI”.

