# Supplementary material for: Targeted mutagenesis of the ryanodine receptor by Platinum TALENs causes slow swimming behaviour in Pacific bluefin tuna (Thunnus orientalis)
Source: Sci Rep. 2019 Sep 25;9:13871. doi: 10.1038/s41598-019-50418-3 (PMC6761128; doi:10.1038/s41598-019-50418-3)
Supplement: Supplementary file 1 — Supplementary Figure [file 41598_2019_50418_MOESM1_ESM.pdf]

Supplementary information for the article:

Targeted mutagenesis of the ryanodine receptor by Platinum TALENs causes slow swimming behaviour in Pacific bluefin tuna (*Thunnus orientalis*)

Kentaro Higuchi[1], Yukinori Kazeto[2], Yuichi Ozaki[3], Toshiya Yamaguchi[2], Yukinori Shimada[2], Yoshiaki Ina[1], Satoshi Soma[1], Yoshitaka Sakakura[4], Rie Goto[5], Takahiro Matsubara[5], Issei Nishiki[6], Yuki Iwasaki[6], Motoshige Yasuike[6], Yoji Nakamura[6], Aiko Matsuura[6], Shukei Masuma[7], Tetsushi Sakuma[8], Takashi Yamamoto[8], Tetsuji Masaoka[3], Takanori Kobayashi[9], Atushi Fujiwara[9], Koichiro Gen[1]

[1]Seikai National Fisheries Research Institute, Japan Fisheries Research and Education Agency, Nagasaki 851-2213, Japan

[2]Kamiura Station, National Research Institute of Aquaculture, Japan Fisheries Research and Education Agency, Saiki, Oita 879-2602, Japan

[3]National Research Institute of Aquaculture, Japan Fisheries Research and Education Agency, Watarai, Mie 519-0423, Japan

[4]Graduate School of Fisheries and Environmental Studies, Nagasaki University, Nagasaki 852-8521, Japan

[5]Nishiura Station, South Ehime Fisheries Research Center, Ehime University, Minamiuwa, Ehime 798-4206, Japan

[6]National Research Institute of Fisheries Science, Japan Fisheries Research and Education Agency, Yokohama, Kanagawa 236-8648, Japan

[7]Aquaculture Research Institute, Kindai University, Nishimuro, Wakayama 649-2211, Japan

[8]Department of Mathematical and Life Sciences, Graduate School of Science, Hiroshima University, Higashi-Hiroshima, Hiroshima 739-8526, Japan

[9]National Research Institute of Fisheries Science, Japan Fisheries Research and Education Agency, Yokohama, Kanagawa 236-8648, Japan

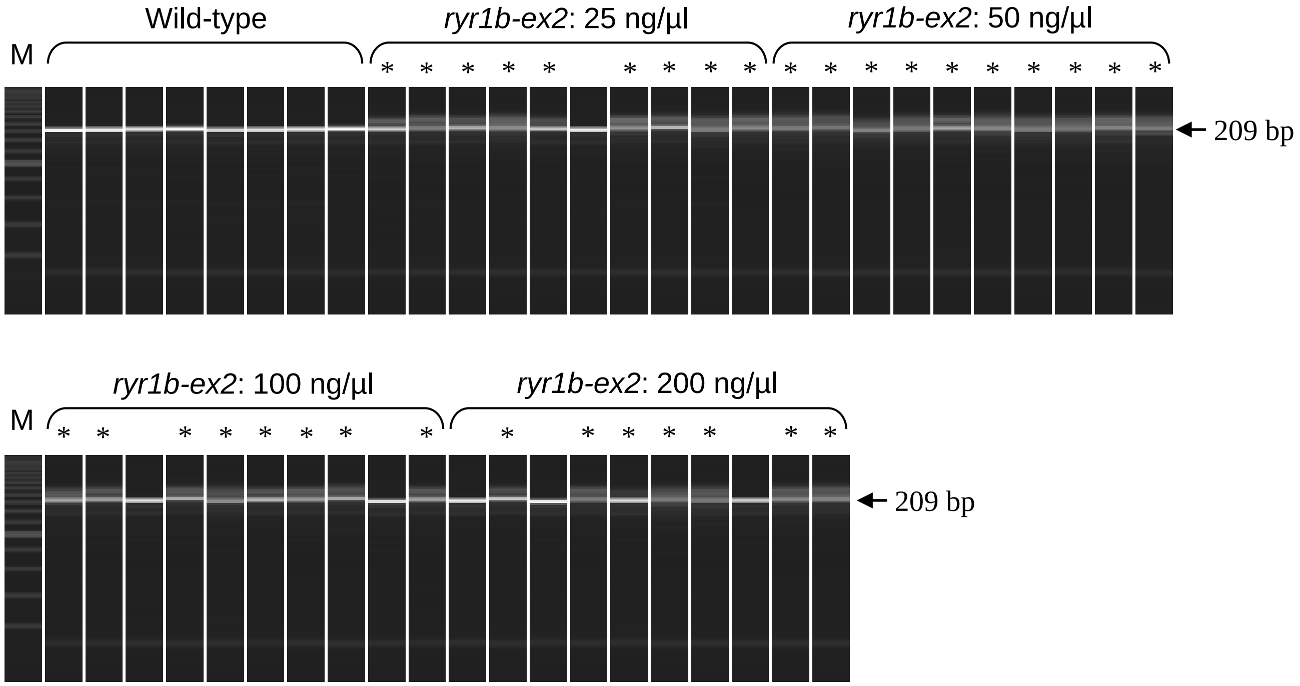

Supplementary Figure S1. The gel images are HMA assays on the representative wild-type and *ryr1b-ex2* TALEN-injected embryos. The upper and lower panels from different gels. Asterisks indicate positive bands from embryos mutated at the targeted sites. M, maker DNA.

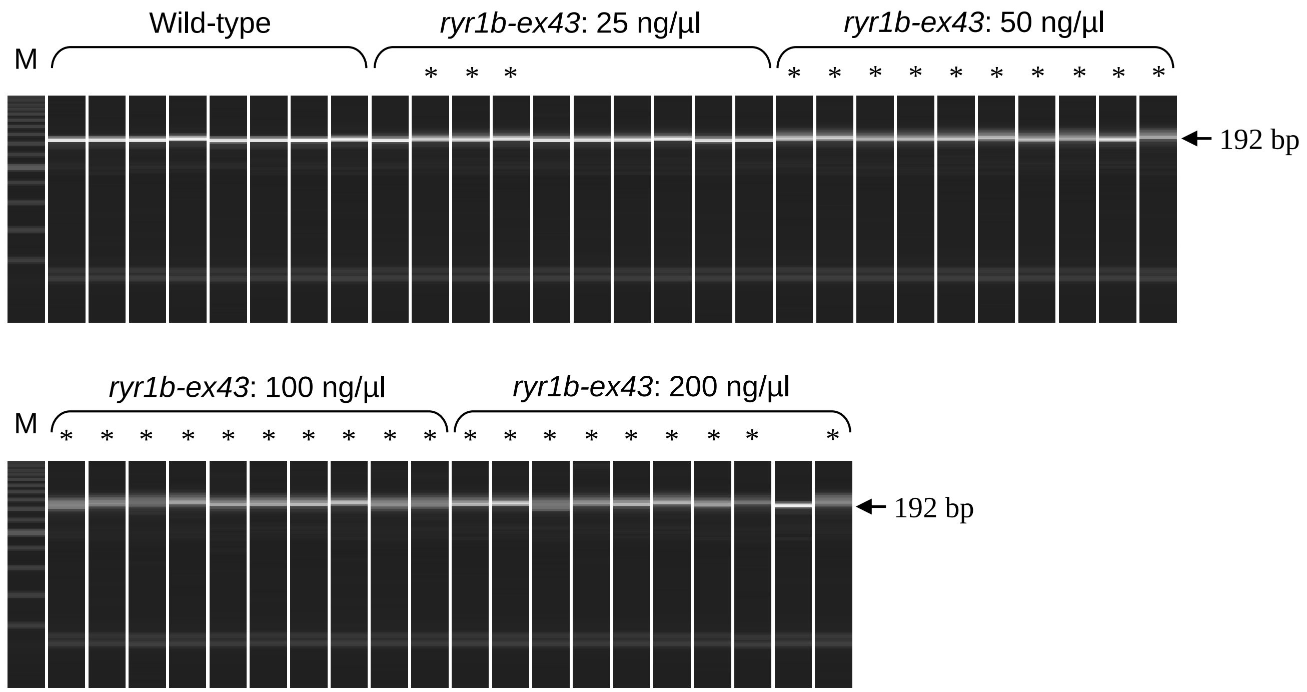

Supplementary Figure S2. The gel images are HMA assays on the representative wild-type and *ryr1b-ex43* TALEN-injected embryos. The upper and lower panels from different gels. Asterisks indicate positive bands from embryos mutated at the targeted sites. M, maker DNA.

ryr1b exon 2 (50 ng/µl)

	Sequence		Frequency (%)
Embryo #1	TATTCTGGAGCAGTCCCTGTCGGTCCGCGCTCTGCAGGAGATGCTGGCCAACACAGTGGAGATGACCGAGGTA	WT	40.4
	TATTCTGGAGCAGTCCCTGTCGGTC-------TGCAGGAGATGCTGGCCAACACAGTGGAGATGACCGAGGTA	-7	17.3
	TATTCTGGAGCAGTCCCTGTCGGTC-----TCTGCAGGAGATGCTGGCCAACACAGTGGAGATGACCGAGGTA	-5	5.7
	TATTCTGGAGCAGTCCCTGTCG-------CTCTGCAGGAGATGCTGGCCAACACAGTGGAGATGACCGAGGTA	-7	4.3
	TATTCTGGAGCAGTCCCTGTCGGTCCGC-------AGGAGATGCTGGCCAACACAGTGGAGATGACCGAGGTA	-7	3.3
Embryo #2	TATTCTGGAGCAGTCCCTGTCGGTCCGCGCTCTGCAGGAGATGCTGGCCAACACAGTGGAGATGACCGAGGTA	WT	41.5
	TATTCTGGAGCAGTCCCTGTCGGTC-------TGCAGGAGATGCTGGCCAACACAGTGGAGATGACCGAGGTA	-7	18.3
	TATTCTGGAGCAGTCCCTGTCGGTC-----TCTGCAGGAGATGCTGGCCAACACAGTGGAGATGACCGAGGTA	-5	5.0
	TATTCTGGAGCAGTCCCTGTCG-------CTCTGCAGGAGATGCTGGCCAACACAGTGGAGATGACCGAGGTA	-7	3.6
	TATTCTGGAGCAGTCCCTGTCGGTCC-----CTGCAGGAGATGCTGGCCAACACAGTGGAGATGACCGAGGTA	-5	2.1
Embryo #3	TATTCTGGAGCAGTCCCTGTCGGTCCGCGCTCTGCAGGAGATGCTGGCCAACACAGTGGAGATGACCGAGGTA	WT	43.3
	TATTCTGGAGCAGTCCCTGTCGGTC-------TGCAGGAGATGCTGGCCAACACAGTGGAGATGACCGAGGTA	-7	9.0
	TATTCTGGAGCAGTCCCTGTCGGTC-----TCTGCAGGAGATGCTGGCCAACACAGTGGAGATGACCGAGGTA	-5	6.7
	TATTCTGGAGCAGTCCCTGTCGG------------------------CCAACACAGTGGAGATGACCGAGGTA	-24	6.0
	TATTCTGGAGCAGTCCCTGTCGG------CTCTGCAGGAGATGCTGGCCAACACAGTGGAGATGACCGAGGTA	-6	5.6
Embryo #4	TATTCTGGAGCAGTCCCTGTCGGTCCGCGCTCTGCAGGAGATGCTGGCCAACACAGTGGAGATGACCGAGGTA	WT	45.7
	TATTCTGGAGCAGTCCCTGTCGGTC-------TGCAGGAGATGCTGGCCAACACAGTGGAGATGACCGAGGTA	-7	11.6
	TATTCTGGAGCAGTCCCTGTCGGTC-----TCTGCAGGAGATGCTGGCCAACACAGTGGAGATGACCGAGGTA	-5	6.1
	TATTCTGGAGCAGTCCCTGTCG-------CTCTGCAGGAGATGCTGGCCAACACAGTGGAGATGACCGAGGTA	-7	5.6
	TATTCTGGAGCAGTCCCTGTCGGTCCGC-------AGGAGATGCTGGCCAACACAGTGGAGATGACCGAGGTA	-7	3.7
Embryo #5	TATTCTGGAGCAGTCCCTGTCGGTC-------TGCAGGAGATGCTGGCCAACACAGTGGAGATGACCGAGGTA	-7	27.5
	TATTCTGGAGCAGTCCCTGTCGGTCCGCGCTCTGCAGGAGATGCTGGCCAACACAGTGGAGATGACCGAGGTA	WT	20.2
	TATTCTGGAGCAGTCCCTGTCGGTC-----TCTGCAGGAGATGCTGGCCAACACAGTGGAGATGACCGAGGTA	-5	9.2
	TATTCTGGAGCAGTCCCTGTCGGTCCGC-------AGGAGATGCTGGCCAACACAGTGGAGATGACCGAGGTA	-7	4.2
	TATTCTGGAGCAGTCCCTGTCG-------CTCTGCAGGAGATGCTGGCCAACACAGTGGAGATGACCGAGGTA	-7	3.5

Wild type

Embryo #1	TATTCTGGAGCAGTCCCTGTCGGTCCGCGCTCTGCAGGAGATGCTGGCCAACACAGTGGAGATGACCGAGGTA	WT	98.0
Embryo #2	TATTCTGGAGCAGTCCCTGTCGGTCCGCGCTCTGCAGGAGATGCTGGCCAACACAGTGGAGATGACCGAGGTA	WT	97.8
Embryo #3	TATTCTGGAGCAGTCCCTGTCGGTCCGCGCTCTGCAGGAGATGCTGGCCAACACAGTGGAGATGACCGAGGTA	WT	98.1

Supplementary Figure S3. Mutant sequences and frequency in the TALEN-targeted site of *ryr1b* exon 2 revealed by sequencing. This is shown in order of sequences, the sizes of deletions (−), and the frequency. WT indicates wild type sequence.

ryr1b exon 43 (200 ng/µl)

			Frequency (%)
Embryo #1	CTGTCCCGCGGCCGAGCCCTTACCAAGGCTCAGCGGGACGTCATTGAGGAGTGCCTCATGTCGCTCTGCAAGT	WT	58.0
	CTGTCCCGCGGCCGAGCCCTTACCAAGGCTCAGCG------------GGAGTGCCTCATGTCGCTCTGCAAGT	-12	6.2
	CTGTCCCGCGGCCGAGCCCTTACCAAGGCTCAGCGGGAC----------AGTGCCTCATGTCGCTCTGCAAGT	-10	3.0
	CTGTCCCGCGGCCGAGCCCTTACCAAGGCTCAGCGGGAC------------AGCCTCATGTCGCTCTGCAAGT	-12	1.8
	CTGTCCCGCGGCCGAGCCCTTACCAAGGCTCAGCGGGAC-------AGACGTGCCTCATGTCGCTCTGCAAGT	-7	1.7
Embryo #2	CTGTCTCGCGGCCGAGCCCTTACCAAGGCTCAGCGGGACGTCATTGAGGAGTGCCTCATGTCGCTCTGCAAGT	WT	71.9
	CTGTCCCGTGGCCGAGCCCTTACCAAGGCTCAGCG------------GGAGTGCCTCATGTCGCTCTGCAAGT	-12	6.4
	CTGTCTCGCGGCCGAGCCCTTACCAAGGCTCAGCG------------GGAGTGCCTCATGTCGCTCTGCAAGT	-12	3.1
	CTGTCCCGTGGCCGAGCCCTTACCAAGGCTCAGCGGGAC-----------GTGCCTCATGTCGCTCTGCAAGT	-11	2.8
	CTGTCTCGCGGCCGAGCCCTTACCAAGGCTCA--------------------GCCTCATGTCGCTCTGCAAGT	-20	1.0
Embryo #3	CTGTCTCGCGGCCGAGCCCTTACCAAGGCTCAGCGGGACGTCATTGAGGAGTGCCTCATGTCGCTCTGCAAGT	WT	11.0
	CTGTCTCGCGGCCGAGCCCTTACCAAGGCTCAGCGGGAC------GAGGAGTGCCTCATGTCGCTCTGCAAGT	-6	8.0
	CTGTCTCGCGGCCGAGCCCTTACCAAGGCTCAGCG------------GGAGTGCCTCATGTCGCTCTGCAAGT	-12	7.9
	CTGTCCCGTGGCCGAGCCCTTACCAAGGCTCAGCGGGAC-----------GTGCCTCATGTCGCTCTGCAAGT	-11	7.2
	CTGTCTCGCGGCCGAGCCCTTACCAAGGCTCAGCGGGAC-----------GTGCCTCATGTCGCTCTGCAAGT	-11	5.5
Embryo #4	CTGTCCCGTGGCCGAGCCCTTACCAAGGCTCAGCGGGACGTCATTGAGGAGTGCCTCATGTCGCTCTGCAAGT	WT	86.1
	CTGTCCCGTGGCCGAGCCCTTACCAAGGCTCAGCGGGA---------------CCTCATGTCGCTCTGCAAGT	-15	1.7
	CTGTCCCGTGGCCGAGCCCTTACCAAGGCTCAGCGGGACGTC-------AGTGCCTCATGTCGCTCTGCAAGT	-7	0.9
	CTGTCCCGTGGCCGAGCCCTTACCAAGGCTCAGCGGGACGT------GGAGTGCCTCATGTCGCTCTGCAAGT	-6	0.9
	CTGTCCCGTGGCCGAGCCCTTACCAAGGCTCAGCGGGAC-----------GTGCCTCATGTCGCTCTGCAAGT	-11	0.8
Embryo #5	CTGTCCCGTGGCCGAGCCCTTACCAAGGCTCAGCGGGACGTCATTGAGGAGTGCCTCATGTCGCTCTGCAAGT	WT	86.5
	CTGTCCCGTGGCCGAGCCCTTACCAAGGCTCAGCG------------GGAGTGCCTCATGTCGCTCTGCAAGT	-12	1.7
	CTGTCCCGTGGCCGAGCCCTTACCAAGGCTCAGC----------------GTGCCTCATGTCGCTCTGCAAGT	-16	1.0
	CTGTCCCGTGGCCGAGCCCTTACCAAGGCTCAGCGGGACGTC----GGGAGTGCCTCATGTCGCTCTGCAAGT	-4	0.7
	CTGTCCCGTGGCCGAGCCCTTACCAAGGCTCAGCGGGACGTCAT----GAGTGCCTCATGTCGCTCTGCAAGT	-4	0.7

Wild type

Embryo #1	CTGTCCCGCGGCCGAGCCCTTACCAAGGCTCAGCGGGACGTCATTGAGGAGTGCCTCATGTCGCTCTGCAAGT	WT	97.6
Embryo #2	CTGTCCCGCGGCCGAGCCCTTACCAAGGCTCAGCGGGACGTCATTGAGGAGTGCCTCATGTCGCTCTGCAAGT	WT	55.7
	CTGTCCCGTGGCCGAGCCCTTACCAAGGCTCAGCGGGACGTCATTGAGGAGTGCCTCATGTCGCTCTGCAAGT	WT	41.8
Embryo #3	CTGTCTCGCGGCCGAGCCCTTACCAAGGCTCAGCGGGACGTCATTGAGGAGTGCCTCATGTCGCTCTGCAAGT	WT	57.0
	CTGTCCCGCGGCCGAGCCCTTACCAAGGCTCAGCGGGACGTCATTGAGGAGTGCCTCATGTCGCTCTGCAAGT	WT	40.6

Supplementary Figure S4. Mutant sequences and frequency in the TALEN-targeted site of *ryr1b* exon 43 revealed by sequencing. This is shown in order of sequences, the sizes of deletions (−), and the frequency. WT indicates wild type sequence. Single nucleotide polymorphisms (SNPs) are underlined.

Supplementary table S1. PCR primer sequences for *ryr1a*, *ryr1b* and *actinb* genes, and PCR product sizes.

Gene	Type for PCR	Primer sequence (5'→3')		Product size (bp)
ryr1a	RT-PCR	Forward primer:	TAATGGAGAGGAGGTGCATC	383
		Reverse primer:	GTTCAACAGGTGCCTCAACC	
	in situ hybridisation	Forward primer:	GTGGTGGCTTTGTCCACTTT	1,178
		Reverse primer:	GAGCTGTCTGGTTCCTCCTG	
ryr1b	RT-PCR	Forward primer:	TGCAGACTTCCTGACAAGTG	576
		Reverse primer:	TCAGGTTCAGCCTTTTCTCC	
	in situ hybridisation	Forward primer:	AATGACGGTGAAGGAGTTGG	1,041
		Reverse primer:	TTGTAGCCGATGATGCAGAG	
actinb	RT-PCR	Forward primer:	GCGACCYCACAGACTACCTC	410
		Reverse primer:	GGCTGTGATCTCCTTCTGCA	

Supplementary table S2. HMA PCR primer sequences for *ryr1b* gene.

Targeted gene site	Primer sequence (5'→3')		Product size (bp)
ryr1b exon 2	Forward primer:	GACTTGGCCATATGCACCTT	209
	Reverse primer:	ACTGAAGCACCTACGCAATC	
ryr1b exon 43	Forward primer:	CGCTACTTGTGTTCAGCTGT	192
	Reverse primer:	TAGTACTGACTTGCAGAGCG	

Supplementary table S3. Quantitative PCR primer sequences for *ryr1b* gene, and PCR product sizes.

Targeted gene site	Primer sequence (5'→3')		Product size (bp)
ryr1b exon 2	Forward primer:	CTGTCGGTCCGCGCTC	84
	Reverse primer:	TAAGGTACGATGGCCTCCAC	
ryr1b exon 43	Forward primer:	AGCGGGACGTCATTGAGG	81
	Reverse primer:	GCCTTCTGAGCAGATGCTGT	

Supplementary Movie S1. Touch-evoked escape behaviour assay on *ryr1b-ex43* mutated Pacific bluefin tuna larvae. The mutated larvae at 7 days after hatching show impaired response to touch.

Supplementary Movie S2. Touch-evoked escape behaviour assay on wild-type Pacific bluefin tuna larvae. The wild-type larvae at 7 days after hatching swim away very rapidly in response to touch.
